# Reliability of EEG in brain death diagnosis: an analysis of clinical and technical factors

**DOI:** 10.3389/fmed.2026.1844133

**Published:** 2026-05-29

**Authors:** Manuel Hinsberger, Adam Strzelczyk, Michael Strüber, Florian J. Raimann, Felix Rosenow, Laurent M. Willems

**Affiliations:** 1 Epilepsy Center Frankfurt Rhine-Main, Department of Neurology, University Hospital, Goethe University Frankfurt, Frankfurt am Main, Germany; 2 Department of Anesthesiology, Intensive Care Medicine and Pain Therapy, University Hospital, Goethe University Frankfurt, Frankfurt am Main, Germany

**Keywords:** anoxic brain injury, electroencephalography, hypoxic brain injury, intensive care, neurocritical care, organ donation, resuscitation

## Abstract

**Introduction:**

In brain death determination (BDD), electroencephalography (EEG) is a commonly used diagnostic method in Germany for proving irreversibility of previously clinically proven loss of brain function. Despite the comprehensive availability of EEG in intensive care units, the reliability of EEG readings is often limited by biological or technical artefacts. This study aimed to determine the detection rate of electrocerebral inactivity (ECI) with a special focus on reliability and challenges of using EEG in BDD.

**Methods:**

A retrospective monocentric analysis of all BDD EEG data acquired from January 2015 to December 2025 at our tertiary care hospital was conducted. All identified BDD EEG reports were systematically analysed for their evaluability and for technical and biological artefacts. In addition, sociodemographic and patient-specific parameters that could influence the accuracy of EEG readings (e.g., craniotomies or intracranial drainages) were recorded.

**Results:**

A total of 47 BDD EEG reports from 42 patients (women: 38.1%, mean age: 48.4 ± 14.4 years, median age: 52 years, age range: 21–78 years) were identified. In 81.0% of the patients, BDD could be confirmed using EEG. While biological artefacts did not significantly limit the evaluability of the EEG readings (*p* > 0.05), technical artefacts were significantly associated with a higher risk for limited EEG readability (*p* = 0.0309, relative risk: 4.2, 95% confidence interval: 1.3–13.8). This finding was primarily attributable to electrode artefacts (*p* = 0.0030), while other artefacts such as electrocardiogram or ventilation artefacts did not significantly impair readability (*p* > 0.05). Neither the aetiology of brain damage nor other patient-specific or sociodemographic parameters had any influence on the usability of EEG in BDD.

**Discussion:**

We conclude that EEG is a reliable diagnostic tool in BDD in most cases if technically accurately recorded. Further studies are required to optimise existing technical errors in selected patients to increase the already high level of methodological reliability.

## 1 Introduction

According to the German Medical Association, electroencephalography (EEG) plays an important supplementary role in brain death determination (BDD) in the context of organ donation or in the assessment of death prior to withdrawal of life-sustaining therapies. In contrast to many other countries, the concept of organ donation after circulatory death has not yet been established in Germany. Donation after brain death or BDD to determine individual death is based on a two-step process consisting of at least one structured clinical examination to determine brain dysfunction and either a further clinical examination at a defined time interval or additional diagnostic testing to prove that the dysfunction is irreversible. The sequence of examinations and the eligibility for all or only certain additional diagnostic procedures are dependent on age and the pattern of damage present (1–3).

As an additional diagnostic tool in BDD, EEG is established and frequently used due to its broad and bedside availability. Based on the German recommendations for BDD, brain death can be confirmed through an EEG reading showing electrocerebral inactivity (ECI) defined as proof of isoelectric EEG (<2 μV) in a special BDD montage with double electrode distances (≥10 cm) and stringent technical and operational requirements (e.g., electrode impedances of <10 kΩ, lower cut-off frequency of 0.53 Hz, upper cut-off frequency of 70 Hz and at least 30 min of artefact-free recording). In addition, early brainstem auditory evoked potentials, somatosensory evoked potentials and cerebral circulatory arrest detected on ultrasound, computed tomography angiography, scintigraphy or digital subtraction angiography are also permitted as additional diagnostic parameters (1, 4, 5). Although computed tomography angiography is increasingly being used due to its methodological reliability (5, 6), BDD through EEG offers several advantages, including its wide availability, standardised evaluation and approved use in patients with mechanical circulatory support devices (3, 7).

Methodological and clinical limitations of the use of EEG in BDD arise from its high susceptibility to confounding factors such as sedatives, narcotics, intoxication, metabolic derailment and hypothermia, which can lead to reversible suppression of EEG activity and promote false-positive findings. In addition, EEG is markedly susceptible to biological and technical artefacts, which are quite common in intensive care units (ICUs) (1–3, 5, 8). In particular, proximity to technical equipment such as syringe or feeding pumps, monitors and mechanical ventilators can cause continuous, intermittent or phasic artefacts that can delay or limit BDD using EEG (1, 4–6). Decompressive craniectomies, intracranial electrodes, drainages and skull fractures can also alter the conduction conditions, thus complicating EEG interpretation (9, 10). Despite these challenges, EEG is feasible in the ICU (11, 12) and a powerful tool that yields faster results than other tests while offering excellent evaluability (13).

**Figure 1:**
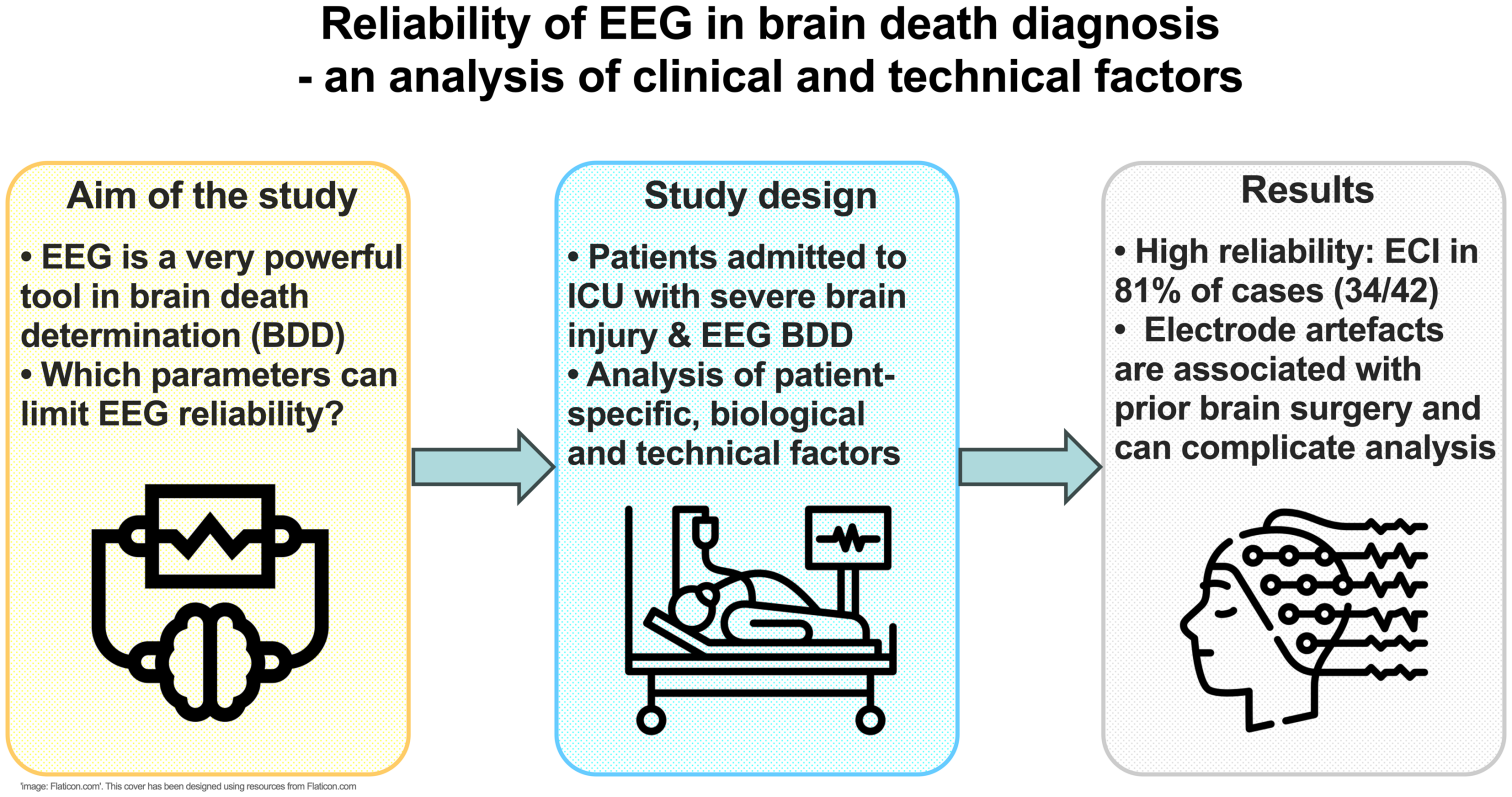
Visual Summary of the study findings on reliability of EEG in brain death diagnosis. Left and right part: Citation: title=”eeg icons”>Eeg icons created by Freepik - Flaticon; License: Flaticon Free License (with attribution); Source: https://www.flaticon.com/free-icons/eeg. Middle part: Citation: title=”icu icons”>Icu icons created by Awicon - Flaticon; License: Flaticon Free License (with attribution); Source: https://www.flaticon.com/free-icons/icu.

The aims of this monocentric retrospective analysis were to evaluate the detection rate of electrocerebral inactivity (ECI), the reliability of EEG in the context of BDD and identify technical, methodological, sociodemographic and patient-specific factors that could influence EEG evaluation and limit its accuracy (Figure 1).

## 2 Materials and methods

### 2.1 Hypothesis

The primary hypothesis of this retrospective evaluation was that the reliability of EEG in the context of BDD may be limited by specific patient-related (e.g., craniotomy/trepanation), biological (e.g., muscle artefacts) and technical factors (e.g., electrical implants). As a central outcome parameter, we have determined the detection rate of electrocerebral inactivity (ECI).

### 2.2 Study design

This study was based on a retrospective analysis of all patients who underwent EEG for BDD diagnostics from 1 January 2015 to 31 December 2025. Patients were identified based on a structured search in the hospital information system of our tertiary care hospital. The EEG data used in our study have not yet been published, as they were not included in previous analyses (14). All BDD EEG data were recorded using a specific report form, which can be automatically retrieved from the hospital information system. Frankfurt University Medical Centre offers comprehensive, highly specialised interdisciplinary care in all medical fields, with a special focus on intensive and neurocritical care (15–18). The STROBE guidelines were followed closely (19), including careful definition of study variables, transparent reporting of inclusion and exclusion criteria, standardised data collection procedures and comprehensive documentation of statistical methods to ensure reproducibility and minimise reporting bias.

Patients were admitted to our ICU following initial work-up, where treating physicians suspected irreversible brain death on the basis of three criteria: (a) acute severe primary or secondary brain damage, (b) pathological clinical signs (coma, brainstem areflexia, or apnoea), and (c) absence of clinical improvement over an appropriate observation period or indication for confirmatory testing. Brain death was confirmed when all three criteria were met. EEG for BDD may be performed in Germany for primary supratentorial, primary infratentorial, or secondary brain damage in patients aged three years or older. When EEG is selected as the confirmatory method, no other ancillary tests are conducted (e.g., brainstem auditory evoked potentials, somatosensory evoked potentials, transcranial Doppler ultrasonography, CT angiography, scintigraphy, or digital subtraction angiography) (1, 4, 5). Accordingly, our study included patients undergoing brain death determination by EEG exclusively, in accordance with German guidelines (1–3).

All EEG recordings met the required technical acceptance criteria as specified by current standards. Each recording was independently reviewed by two experienced specialists as part of the routine diagnostic work-up, ensuring consistent interpretation. Subsequently, all EEGs were analysed for the presence of artefacts and classified according to established guidelines (20, 21).

All identified adult patients (≥18 years of age at the time of BDD) with at least one EEG reading for BDD were consecutively included in the analysis. Informed consent was neither possible nor needed, since all patients included had already passed away at study entry. This study was approved in advance by the local ethics committee of Frankfurt University Hospital. No ethical concerns were raised regarding its implementation (local reference number 2026-2783).

The following variables were manually extracted from the hospital information system and analysed in a pseudonymised manner:

1. Sociodemographic and BDD-related variables: age, sex, pre-existing medical conditions, cause of acute brain damage, performed or previous skull surgery, size of craniectomy (burr-hole trepanation vs. hemicraniectomy), length of stay in the ICU, time from admission to EEG recording and BDD, result of BDD, i.e., ECI (present vs. absent or unreliable EEG), and organ procurement following BDD.
2. EEG-related variables: time of admission, time of suspected brain death, date and duration of recording, artefacts (e.g., electrocardiogram [ECG], pulse, electrode, muscle, ventilator or extraneous artefacts), limitations of accessibility according to EEG reports and comments and success of BDD through EEG.

Artefacts were defined and classified according to Tatum et al. (20) as follows. *ECG artefacts* are characterised by QRS-like spikes occurring predominantly in temporobasal regions (e.g., ear electrodes), triggered by the cardiac electric field. *Pulse artefacts* present as lateralised or diffuse rhythmic slow waves following the QRS complex, typically time-locked with a delay of approximately 200 milliseconds. *Electrode artefacts* manifest as complex repetitive discharges resulting from faulty electrical connections at the electrode–skin interface or anywhere along the recording circuit. *Muscle artefacts* arise from activity of common muscles (e.g., temporalis or frontalis) and are characterised by high-frequency spike components with a duration of less than 20 milliseconds, reflecting underlying motor unit potentials. *Ventilator artefacts* appear as slow periodic deflections at a rate of 12–20 per minute, corresponding to ventilator-associated body movements. *Extraneous artefacts* represent external electrical noise from environmental sources, including infusion pumps and intravenous drip systems, which may generate capacitive, inductive, or electrostatic interference (20).

All EEG recordings were independently reviewed by two experienced specialists. In a first step, each recording was assessed for the presence of biological artefacts (ECG, pulse, muscle) and technical artefacts (ventilator, electrode, extraneous). If no artefacts were identified, the recording was classified as artefact-free, and a definitive interpretation regarding electrocerebral inactivity (ECI)—either confirmed or not confirmed—was rendered. If artefacts were present, the interpreting specialists additionally assessed whether a conclusive ECI determination remained possible despite artefact interference. Recordings in which artefacts precluded a definitive ECI interpretation were classified as “unreliable”, whereas recordings in which ECI could be conclusively confirmed or excluded despite the presence of artefacts were retained as interpretable or “reliable”. The primary aim of this analytical framework was to evaluate whether the presence of artefacts per se is associated with reduced EEG interpretability, and whether this association differs depending on the type of artefact encountered.

### 2.3 Statistical analysis

Statistical analyses were performed using GraphPad Prism v11.0.0 (GraphPad Software, Boston, MA, US). Normality was tested using the D’Agostino–Pearson omnibus test. Normally distributed data were presented as means ± SDs and analysed using Student’s t-test or ANOVA. Non-parametric data were analysed using the Mann–Whitney test or the Kruskal–Wallis test. Categorical variables were compared using Fisher’s exact test. Holm–Bonferroni correction was applied for multiple comparisons. A *p*-value of <0.05 was considered statistically significant.

## 3 Results

### 3.1 Patient cohort

A total of 47 BDD EEG recordings from 42 patients were examined. While one EEG was sufficient as part of BDD in most patients (90.5%), three patients (9.3%) underwent two EEGs, and one patient had three EEGs. The mean patient age was 48.4 ± 14.4 years (median: 52 years, range: 21–78 years), and approximately 38.1% were women. The mean time from admission to EEG recording was 4.2 ± 3.88 days (median: 3.1, range: 0.6–16.8) and the mean time from clinically suspected brain death to EEG recording was 228 ± 274 min (median: 151, range: 12–1,515). Further information on the sociodemographic, disease-related and BDD-specific aspects is shown in Table 1. In order to address potential intra-subject correlation, the further analysis is restricted to the first EEG per patient (*n* = 42), while the remaining 5 repeated measurements were excluded.

**Table 1:** Sociodemographic and disease-specific patient characteristics.

Variable	Parameter	% (*n*)
Age, year	Mean ± SD	48.4 ± 14.4
	Median	52
	Range	21–78
Sex	Female	38.1 (16)
	Male	61.9 (26)
Underlying disease	Hypoxic-ischaemic encephalopathy	38.1 (16)
	Subarachnoid haemorrhage	31.0 (13)
	Intracerebral haemorrhage	14.3 (6)
	Malignant ischaemic stroke	9.5 (4)
	Traumatic brain injury	4.8 (2)
	Cerebral sinus-vein thrombosis	2.4 (1)
Mechanism of brain damage	Primary	78.6 (33)
	Secondary (hypoxic)	21.4 (9)
Location of brain damage	Supratentorial	64.3 (27)
	Infratentorial	11.9 (5)
	Combined	23.8 (10)
EEG recordings for BDD	One EEG recording	90.5 (38)
	Multiple EEG recordings	9.5 (4)
	*n* = 2	7.1 (3)
	*n* = 3	2.4 (1)
Time from admission to EEG recording (days)	Mean ± SD	4.2 ± 3.9
	Median	3.1
	Range	0.6–16.8
Time from clinically suspected brain death to EEG recording (minutes)	Mean ± SD	228 ± 274
	Median	151
	Range	12–1,515
Electrocerebral inactivity	Confirmed	81.0 (34)
	Not confirmed	4.8 (2)
	Assessment not possible	14.3 (6)
Brain surgery prior to EEG	Performed	52.4 (22)
	Burr-hole trepanation	38.1 (16)
	Hemicraniectomy	33.3 (14)
	Skull fracture repair	11.9 (5)
	Not performed	47.6 (20)
Superimposed artefact	Reported	71.4 (30)
	Not reported	28.6 (12)
Organ donation after BDD (*n* = 34)	Desired	73.5 (25)
	Declined	26.5 (9)

### 3.2 Reliability of EEG in BDD – outcome analysis

The diagnosis of brain death according to EEG criteria was feasible in 34 patients (81.0% with ECI). The initial EEG showed cerebral electrical activity in 9.5% of the patients (4/42), whereas a second recording after 34.9 ± 15.7 h confirmed brain death in one patient (2.4%; 1/42); brain death was ruled out through EEG in another patient (2.4%; 1/42). In eight patients, brain death could not be confirmed in the initial EEG due to biological and technical reasons. Conversely, one patient and another patient needed a second EEG and two consecutive EEGs to confirm the diagnosis of brain death, respectively. The mean delay time was 21.7 ± 0.9 h. There were six patients (14.3%; 6/42) in whom brain death analysis via EEG was not possible due to artefact interference.

We investigated which artefacts occurred in the 42 EEG analyses and how they affected the reliability of our analysis (i.e., whether EEG could distinguish electrical activities/ECIs from artefacts). In 34 EEGs (81.0%), specialists noted the presence of superimposed artefacts in the submitted EEG recordings. Our detailed analysis revealed two classes: biological (i.e., ECG, pulse and muscle artefacts) and technical artefacts (i.e., ventilator, electrode and external artefacts) (Table 2).

**Table 2:** Impact of the sociodemographic and disease-related variables on EEG reliability.

Variable	Reliable EEG (*n* = 34, % [*n*])^§^	Unreliable EEG (*n* = 8)^§^	Univariate *p*-value^*^	Holm-adjusted *p*-value^#^
Age, year	50.4 ± 14.0	41 ± 15.4	0.1019	0.9171
Sex, female	38.2 (13)	37.5 (3)	0.9716	>0.9999
Any biological artefacts	67.6 (23)	100.0 (8)	0.0820	0.8200
Electrocardiogram	58.8 (20)	75.0 (6)	0.6879	>0.9999
Pulse	47.0 (16)	62.5 (5)	0.6965	>0.9999
Muscle	2.9 (1)	12.5 (1)	0.3484	>0.9999
Burr-hole trepanation	32.4 (11)	62.5 (5)	0.2233	>0.9999
Craniectomy	32.4 (11)	37.5 (3)	>0.9999	>0.9999
Any technical artefacts	20.6 (7)	62.5 (5)	**0.0309**	0.3399
Electrode	8.8 (3)	62.5 (5)	**0.0030**	**0.0360**
Ventilator	8.8 (3)	0.0(0)	>0.9999	>0.9999
Others	17.6 (6)	12.5 (1)	>0.9999	>0.9999

^§^Analysis was restricted to the first EEG per patient (*n* = 42) to exclude potential within-patient material from multiple recordings (*n* = 5). ^*^The *p*-values were compared between the reliable and unreliable EEG groups after testing for normality. Either Student’s *t*-test or Welch’s test was performed for the group comparison and Fisher’s exact test for the identification of risk factors. ^#^Holm–Bonferroni correction was applied for multiple comparisons. Values are shown as proportions in percentages with total numbers in brackets. Bold values indicate significant results, *p* < 0.05.

### 3.3 Reliability of EEG in BDD – clinical parameters

We did not detect any relevant significant trends (*p* > 0.05) for primary/secondary brain injury, traumatic brain injury or infra−/supratentorial brain injury in the EEG reliability analyses. Additionally, there was no significant influence (*p* > 0.05) of any disease on the probability of biological or technical artefacts.

### 3.4 Reliability of EEG in BDD – biological and technical artefacts

**Figure 2:**
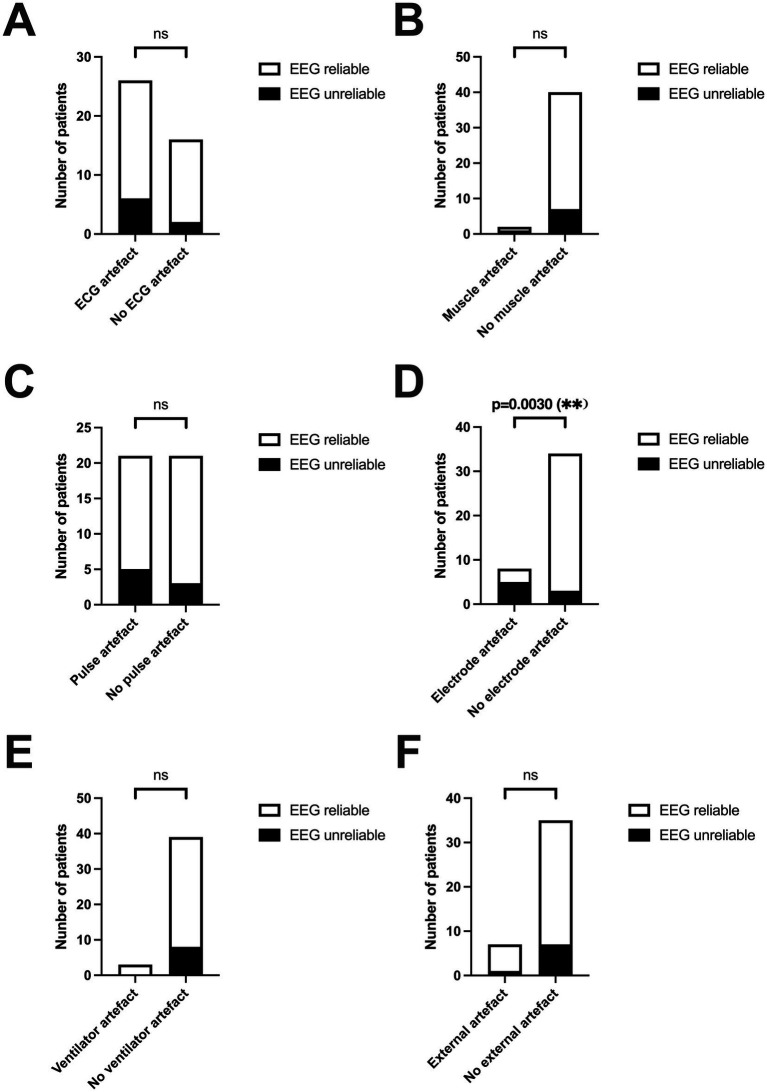
Evaluation of biological and technical artefacts relative to EEG reliability **(A–F)**. In all 42 patients, EEG (electroencephalography) reliability (white) and unreliability (black) were compared across different artefacts. Nominal values and statistical comparison are shown, with the significance level set at *p* < 0.05. ns = not significant, ^**^0.001 ≤ *p* < 0.01.

Biological artefacts occurred in all 33 artefact-superimposed EEGs. Most of them were ECG artefacts (28/33; 84.9%), followed by pulse (23/33; 69.7%) and muscle artefacts (2/33; 6.1%). When these biological artefacts occurred, they did not lead to a significant increase in EEG unreliability (Figures 2A–C).

In contrast, technical artefacts were less frequently observed (15/33; 45.5%), and when they occurred, there was a significant trend (*p* = 0.0309) towards EEG unreliability (relative risk: 4.2; confidence interval [CI]: 1.3–13.8). In particular, electrode artefacts were found to be a significant factor (*p* = 0.0030) associated with EEG unreliability (Figure 2D). Fisher’s exact test revealed that the presence of electrode artefacts (compared with their absence) was not only significant but also associated with a markedly increased risk of an EEG being classified as unreliable rather than reliable (relative risk: 7.1; 95% CI: 2.2–22.3). Conversely, both ventilator and external artefacts were not significantly (*p* > 0.05) associated with greater unreliability of EEG interpretation (Figures 2E,F).

After post-hoc correction for multiple testing using Holm–Bonferroni correction (*α* = 0.05), only the presence of electrode artefacts remained significantly associated with an impaired readability of EEG records. All other factors remained non-significant (Table 2).

**Figure 3:**
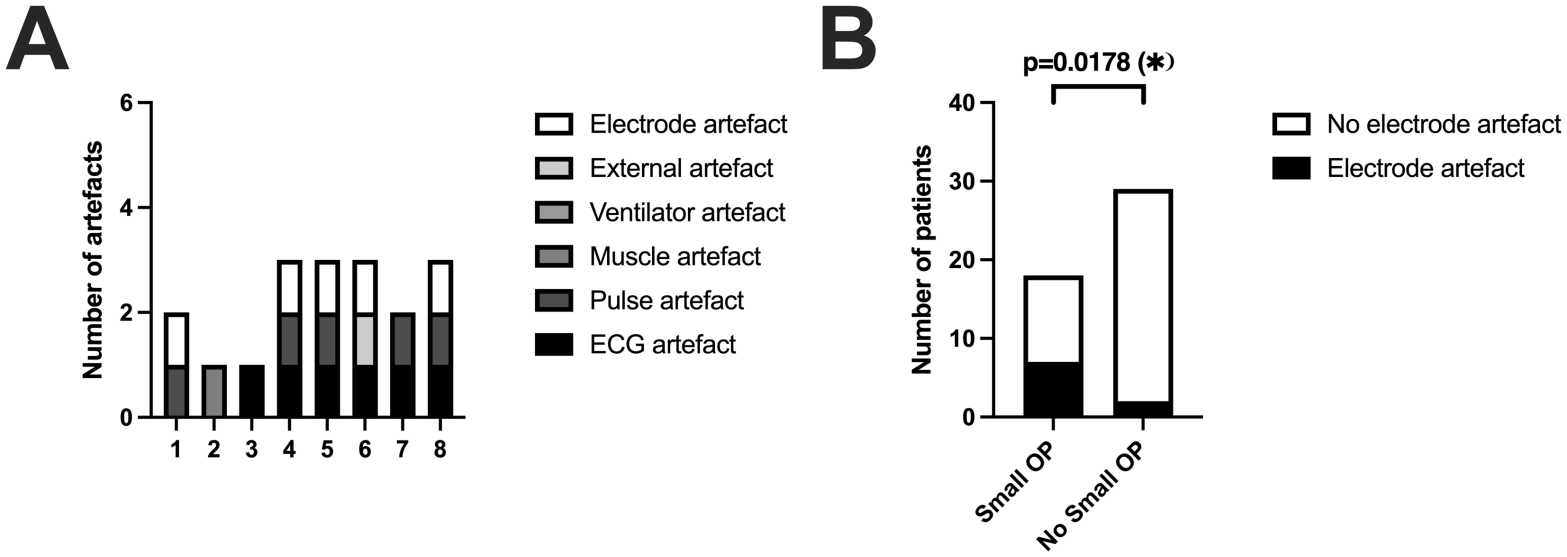
Detailed analyses among the patients with unreliable EEG readings: **(A)** Subdivision of several artefacts in the cohort of eight unreliable EEG (electroencephalography) readings. Electrode (white), external (brighter grey), ventilator (bright grey), muscle (grey), pulse (dark grey), and ECG artefacts (black) were listed in groups according to their appearance in each patient. These EEGs were obtained from distinct patients. **(B)** In all 42 patients, the presence of electrode artefacts was determined and categorised as ‘yes’ (black) or ‘no’ (white). These groups were then compared based on whether the patients underwent a small OP (left) or not (right). Nominal values and statistical comparison are shown, with the significance level set at *p* < 0.05. ns = not significant, ^*^0.01 ≤ *p* < 0.05.

We also analysed the factors potentially associated with a higher frequency of electrode artefacts, since these are the most common and clinically relevant technical artefacts contributing to EEG unreliability (Figure 3A). In this context, it is important to note that those eight EEGs were performed on different patients. We detected a significant trend in the patients who underwent a small operative procedure (relative risk: 5.6; CI: 1.5–22.2) (*p* = 0.0178) (Figure 3B) or both small and large operative procedures (relative risk: 5.3; CI: 1.8–15.0) (*p* = 0.0074), a trend that was not observed when only a large operative procedure was performed (*p* = 0.2516). However, no significant trend (*p* > 0.05) was found between the presence of surgical procedures – whether small, large or both – and the unreliability of EEG interpretation.

## 4 Discussion

Over the last decades, EEG has been a central diagnostic tool used in BDD in Germany due to its general availability and almost unrestricted usability across most patient groups with different underlying aetiologies of brain damage (22). Although the guidelines and practices for BDD vary significantly between countries, EEG as part of diagnostics is mandatory in several countries and the most frequently used ancillary test (23). Most available studies have focused on the reliability of EEG in proving or ruling out ECI in critically ill patients (7, 24). Several studies have highlighted the technical limitations of EEG in BDD, particularly with regard to signal quality and artefact interference (15, 20, 25). In this study, we evaluated the reliability of EEG as an ancillary tool for BDD from a methodical view, with a particular focus on the impact of biological and technical artefacts.

In line with previous studies, we found EEG as a reliable ancillary test for confirming BDD in most cases (12). The proof of ECI in 81% of the EEGs in the present study is below previously reported rates in Spanish (97%) and Italian cohorts (100%) (7, 24). Although the BDD protocols for EEG in both countries are similar to those in Germany (26), neither study explicitly addresses potential artefacts that could limit the analysability and reliability of EEG recordings. Even though the cardiopulmonary function of patients with irreversible brain damage can often be stabilised after initial autonomic dysregulation in the ICU, this condition is highly vulnerable, and death is possible at any time. In a recent study, BDD could not be completed in approximately 10% of patients due to haemodynamic instability during death or before workup completion (27). Particularly in terms of optimal resource allocation and rapid and effective BDD, the technical or biological limitations of certain ancillary tools in the prognostic pathway are highly relevant.

The present analysis therefore focused primarily on cases in which ECI-based diagnosis was plausible but formally unsuccessful due to artefacts. As mentioned previously, artefacts encountered in the ICU during continuous or routine EEGs are more complex and may have relevant therapeutic implications compared with those in other more standardised recording settings (e.g., outpatient units or clinical EEG laboratories) (28). In the present cohort, 70% of the EEGs were annotated as superimposed by technical or biological artefacts (Figure 3A and Table 2) (20, 21). The subsequent analyses focused on the potential clinical implications of these artefacts. Given the relatively small number of technically unreliable EEG recordings, the results should be interpreted as exploratory rather than confirmatory, as reflected by the widened confidence intervals throughout. While biological artefacts such as pulse, ECG or muscle artefacts were described much more frequently than technical artefacts, their effect on the readability of the EEG recordings was not relevant in the context of BDD. Burr-hole trepanations and craniotomies, regardless of their size, which usually lead to relevant breach rhythms and asymmetries (29), were not associated with reduced EEG reliability in BDD. This is primarily due to the familiarity of biological artefacts to experienced EEG diagnosticians (30). In contrast, many technical artefacts in the ICU, such as ventilator, pump or other external artefacts, are unfamiliar (30). Electrical artefacts are also difficult to correct or shield in the ICU, especially if they involve implanted devices such as pacemakers or if the sources are vital devices that must be in the immediate vicinity of the patient or must be connected to them by cable or line (31). This is particularly challenging for patients with intra- or extracorporeal circulatory support devices, such as percutaneous micro-axial flow pumps or extracorporeal membrane oxygenation. Nevertheless, even in these patients, in whom other perfusion-based ancillary procedures are contraindicated due to low systemic perfusion pressure, BDD via EEG can be achieved through optimised electrical shielding and cable routing (3).

Due to their mostly regular or almost rhythmic occurrence (e.g., respiratory artefacts) and often fixed frequency bands (e.g., electrostatic artefacts), modern approaches to artefact reduction in the ICU represent a promising option for reducing the extent of technical artefacts in this setting and thus improving the evaluability of EEG signals (32). With automatic algorithms based on rapid blind source separation, biological artefacts could be reduced by 80%–98% depending on their origin in a practical setting, and technical powerline artefacts could be completely eliminated from the original EEG signals (33). In addition to automatic algorithms, approaches based on artificial intelligence for improving signal quality and reducing artefacts also represent a promising option (34–38). However, according to current jurisdiction, at least in Germany, the use of any additional filters, algorithms or artificial intelligence tools for artefact reduction is not permitted within the framework of BDD using EEG (1–3).

After successful BDD through EEG, organ donation based on the documented or presumed wishes of the patients was possible in 74% of the cases. However, clear communication of BDD and the use of additional diagnostic tools to technically confirm the irreversible cessation of brain function may help to increase the acceptance of individual death among relatives and thus indirectly improve their willingness.

Since the dataset presented was derived from real-world neurocritical care practice over a period of more than 10 years, we consider its robustness to be a major advantage. However, the retrospective single-centre study design limits generalisability (39). Some sociodemographic factors, national peculiarities, selection bias and inter-observer reliability could influence the study results. We acknowledge that the decision to restrict the primary analysis to the first EEG per patient (*n* = 42) reduces the effective sample size; however, this approach improves the validity of the statistical assumptions by preserving the independence of observations and avoiding within-patient correlation. Notably, the primary findings remained consistent when the full dataset including repeated examinations (*n* = 47) was analysed, supporting the robustness of our results. We attempted to address this by applying the Holm–Bonferroni correction for multiple comparisons, and systematic reporting followed the STROBE guidelines closely to limit these and other methodological biases (19). Nevertheless, with only eight unreliable EEG recordings available, multivariate modelling was not feasible, and the study was insufficiently powered to detect small effect sizes. In addition, caution is warranted if the regulations or brain death protocols—such as those in other countries—do not align with those in our study. These limitations should be considered when interpreting the findings. Consequently, future multicentre studies combining EEG with other ancillary modalities (e.g., cerebral angiography or perfusion imaging) could help to better quantify the diagnostic performance of EEG under varying artefact burdens.

In conclusion, EEG is a widely used, rapid and reliable ancillary diagnostic tool for BDD. While biological artefacts seem to be mostly irrelevant, technical artefacts can significantly limit the interpretability of EEG results. Consequently, attention should be paid to reducing artefacts as much as possible during testing. If artefacts persist and prevent ECI detection, further imaging, electrophysiological or clinical assessments can be used for BDD, depending on the type of brain damage and the applicable national guidelines, which must be considered in such cases.

## Data Availability

The original contributions presented in the study are included in the article/supplementary material, further inquiries can be directed to the corresponding author.
